# Associations and prognostic significance of diffuse myocardial fibrosis by cardiovascular magnetic resonance in heart failure with preserved ejection fraction

**DOI:** 10.1186/s12968-018-0477-4

**Published:** 2018-08-08

**Authors:** Clotilde Roy, Alisson Slimani, Christophe de Meester, Mihaela Amzulescu, Agnes Pasquet, David Vancraeynest, Christophe Beauloye, Jean-Louis Vanoverschelde, Bernhard L. Gerber, Anne-Catherine Pouleur

**Affiliations:** 1 Division of Cardiology, Department of Cardiovascular Diseases, Cliniques Universitaires St. Luc UCL, Av Hippocrate 10/2806, B-1200 Woluwé St. Lambert, Belgium; 2 Pôle de Recherche Cardiovasculaire (CARD), Institut de Recherche Expérimentale et Clinique (IREC), Université Catholique de Louvain, Brussels, Belgium

**Keywords:** Diffuse myocardial fibrosis, Cardiac magnetic resonance, Prognosis

## Abstract

**Background:**

Increased myocardial fibrosis may play a key role in heart failure with preserved ejection fraction (HFpEF) pathophysiology. The study aim was to evaluate the presence, associations, and prognostic significance of diffuse fibrosis in HFpEF patients compared to age- and sex-matched controls.

**Methods:**

We prospectively included 118 consecutive HFpEF patients. Diffuse myocardial fibrosis was estimated by extracellular volume (ECV) quantified by cardiovascular magnetic resonance with the modified Look-Locker inversion recovery sequence. We determined an ECV age- and sex-adjusted cutoff value (33%) in 26 controls.

**Results:**

Mean ECV was significantly higher in HFpEF patients versus healthy controls (32.9 ± 4.8% vs 28.2 ± 2.4%, *P* <  0.001). Multivariate logistic regression showed that body mass index (BMI) (odds ratio (OR) =0.92 [0.86–0.98], *P* = 0.011), diabetes (OR = 2.62 [1.11–6.18], *P* = 0.028), and transmitral peak E wave velocity (OR = 1.02 [1.00–1.03], *P* = 0.022) were significantly associated with abnormal ECV value. During a median follow-up of 11 ± 6 months, the primary outcome (all-cause mortality or first heart failure hospitalization) occurred in 38 patients. In multivariate Cox regression analysis, diabetes (hazard ratio (HR) =1.98 [1.04; 3.76], *P* = 0.038) and hemoglobin level (HR = 0.81 [0.67; 0.98], *P* = 0.028) were significant predictors of composite outcome. The ECV ability to improve this model added significant prognostic information. We then developed a risk score including diabetes, hemoglobin and ECV > 33% demonstrating significant prediction of risk and validated this score in a validation cohort of 53 patients. Kaplan–Meier curves showed a significant difference according to tertiles of the probability score (*P* <  0.001).

**Conclusion:**

Among HFpEF patients, high ECV, likely reflecting abnormal diffuse myocardial fibrosis, was associated with a higher rate of all-cause death and first HF hospitalization in short term follow up.

**Trial registration:**

Characterization of Heart Failure With Preserved Ejection Fraction. Trial registration number: NCT03197350. Date of registration: 20/06/2017. This trial was retrospectively registered.

**Electronic supplementary material:**

The online version of this article (10.1186/s12968-018-0477-4) contains supplementary material, which is available to authorized users.

## Background

Heart failure (HF) with preserved ejection fraction (HFpEF) has been established as a major cause of cardiovascular morbidity and mortality, especially among the elderly [1, 2]. Prevalence is increasing, affecting half of the patients with clinical signs of heart failure [3]. However, compared to HF with reduced ejection fraction (HFrEF), survival in HFpEF has not improved over time, and to date, no treatment effectively improves outcomes, probably because of the phenotypic heterogeneity of this syndrome [4].

Several mechanisms have been implicated in HFpEF, including advanced age and cardiovascular, metabolic, and pro-inflammatory comorbidities such as hypertension, diabetes, obesity, chronic obstructive pulmonary disease), coronary disease and renal failure [1, 4–6]. The exact pathophysiology of HFpEF remains unclear as a result of the absence of a proper animal model and the presence of numerous confounding effects. Recently, several studies using autopsies or myocardial biopsies have highlighted the key role of myocardial extracellular matrix abnormalities (fibrosis [7–9]) and myocardial structural changes such as altered cardiomyocyte function (hypertrophy [7]), systemic and coronary microvascular inflammation, and endothelial dysfunction (oxidative stress) as all being involved in myocardial stiffness and diastolic dysfunction [10, 11].

Quantification of extracellular volume fraction (ECV) by cardiovascular magnetic resonance (CMR) has recently emerged as a novel non-invasive diagnostic tool to assess myocardial fibrosis [12–14]. Recently, some studies have demonstrated the importance of diffuse or focal fibrosis estimated by biopsies or/and by CMR in patients with HFpEF [15, 16].

The aim of our study was to evaluate the presence, associations, and prognostic significance of ECV, likely reflecting diffuse myocardial fibrosis, in HFpEF patients.

## Methods

### Study population

Between December 2014 and October 2016, consecutive patients with suspected HFpEF were prospectively evaluated for inclusion in the study. The local ethics committee approved the study, and all patients gave written informed consent before study enrollment (Clinical trial NCT03197350).

The following criteria had to be fulfilled for study inclusion: New York Heart Association functional (NYHA) class ≥II, typical signs of HF, NT-proBNP > 350 pg/ml and/or a hospitalization for HF in the previous 12 months, left ventricular (LV) ejection fraction (EF) ≥50%, and relevant structural heart disease (LV hypertrophy/left atrial (LA) enlargement) and/or diastolic dysfunction by echocardiography [17]. Ischemic cardiomyopathy was defined as history of myocardial infarction or revascularization by either coronary artery bypass graft surgery (CABG) or coronary artery angioplasty. Diabetes was defined as an abnormal fasting glycaemia (> 126 mg/dl) or the use of antidiabetic drugs.

The exclusion criteria were any contraindications to CMR (pacemaker, estimated glomerular filtration rate (eGFR) < 30 ml/min/m^2^, claustrophobia), severe valvular disease, infiltrative (ie amyloidosis, sarcoidosis or hypertrophic cardiomyopathy, acute coronary syndrome in the last 30 days, chronic obstructive pulmonary disease GOLD 3 or 4, congenital heart disease, pericardial disease, atrial fibrillation with a ventricular response > 140 bpm, and severe anemia (hemoglobin < 7 g/dl). Patients with severe cognitive disorder were also excluded. One patient screened for the study was excluded due to presence of unknown cardiac amyloidosis.

Patients were compared to 26 age- and sex-matched controls without history of cardiovascular disease. Controls were recruited by advertisement in the community. All subjects underwent a full clinical exam, electrocardiogram (ECG), transthoracic echocardiography and exercise stress test, which all had to be normal prior to inclusion.

Controls and patients underwent blood sampling, complete transthoracic echocardiography and a CMR.

### Echocardiography

All subjects underwent a two-dimensional (2D) transthoracic echocardiography at inclusion (iE33 system Philips Healthcare, Best, The Netherlands) with parasternal long- and short-axis views and apical views to assess LV and right ventricular (RV) systolic and diastolic functions and measurements of LA and right atrial (RA) volumes, as well as a valvular evaluation. Mitral valve inflow pattern (E and A velocity) and septal and lateral mitral valve annular velocities (e’) were recorded.

RV function was assessed by systolic annular tissue velocity of the lateral tricuspid annulus, tricuspid annular plane systolic excursion (TAPSE), and tracing the RV endocardium in the apical four-chamber view in systole and diastole to obtain fractional area change (RV FAC%) [18]. All measurements were averaged over three beats in atrial fibrillation.

### Cardiovascular magnetic resonance

CMR was performed using a 3 Tesla system (Ingenia, Philips Hearlthcare). Briefly, after localization of the heart, to assess LV and RV myocardial function and mass, 10–12 consecutive short-axis (SAX) images and 2-, 3-, and 4-chamber long-axis images of the LV were acquired using a cine balanced steady-state free precession sequence (bSSFP). Then, mid-ventricular short-axis modified Look-Locker inversion recovery (MOLLI) images were acquired for T1 determination using an 11-image, 18-heartbeat 3-(3)-3-(3)-5 bSSFP sequence. A total dose of 0.2 mmol/kg gadobutrol (Gadavist, Bayer Healthcare Leverkusen, Germany) was injected, and 10–15 min after contrast injection, short- and long-axis 2D inversion recovery late gadolinium enhancement (LGE) images were acquired to evaluate focal myocardial fibrosis. Finally, 15-min post-contrast, MOLLI T1 mapping was repeated in a protocol identical to that used for pre-contrast T1 mapping. The presence of LGE was visually assessed.

Pre- and post-contrast MOLLI images were processed using the open-source software MRmap v1.4 [19] under IDL. Images were corrected for respiratory motion when needed. T1 maps were exported to Osirix 5.7 (Pixmeo, Geneva, Switzerland) and pre- and post-myocardial T1 times were measured in six regions-of-interest (ROI) in the myocardium (anterior, anterolateral, inferolateral, inferior, inferoseptal, anteroseptal). We calculated the average T1 time of the six different ROIs. Areas of ischemic focal fibrosis identified by late gadolinium enhancement were excluded from the analysis. The partition coefficient lambda (λ) and ECV were then computed according to the formula [20].

End-diastolic and end-systolic LV and RV volumes as well as LV mass and presence of LGE were analyzed using the freely available software Segment 2.0 (Medviso, Lund, Sweden), as previously described. LGE was considered present if myocardial enhancement was observed on both short-axis and long-axis views. The total LGE volume was calculated by summing the LGE volume of all slices, and the ratio (%) of LGE was then calculated using the same software.

### Follow-up

Patients were prospectively followed by ambulatory visits and telephone calls at 6-month intervals. Clinical and survival status was obtained by follow up visits and by phone contact with the patients, their relatives, and their physician if necessarily. The primary outcome was a composite of all-cause mortality or a first hospitalization for HF. Vital status was ascertained by medical record review. First HF hospitalization was defined as patients treated in the emergency room or admitted to a hospital and requiring intravenous diuretics. Patients had at least one symptom and 2 signs of HF (peripheral edema, pulmonary crackles, high NT-proBNP level, radiological signs of pulmonary congestion or hemodynamic evidence).

### Statistical analysis

Statistical analyses were performed using SPSS version 22 (International Business Machines, Inc., Armonk, New York, USA) and STATA version 11 (Stata Corporation, College Station, Texas, USA) software. All tests were two-sided, and a *P* < 0.05 was considered statistically significant. Continuous variables were expressed as mean ± 1 SD if normally distributed or as medians (25th and 75th percentiles) if not normally distributed. Categorical variables were expressed as counts and percentages. Comparison between groups was performed using ANOVA, chi-square test, or unpaired t-tests when appropriate. We determined an ECV age and sex-adjusted cutoff value corresponding to the upper 95% confidence interval of 26 age- and sex matched volunteers (ECV ≥ 33%).

Logistic regression was performed to determine predictors of abnormal diffuse fibrosis (ECV above or below 95% confidence intervals in controls). For this purpose, after univariate comparison of the two groups, parameters with a *P* < 0.10 were proposed for inclusion in the multiple logistic regression analysis with a backward selection procedure.

Event-free survival was estimated using Kaplan–Meier methods and Cox regression analysis. All baseline and imaging variables were initially proposed for inclusion in a univariate Cox proportional hazard model. To avoid colinearity in the Cox regression model, the correlation coefficients between covariates were examined. In cases of colinearity (*r* > 0.50), only the strongest of the two covariates was proposed for inclusion into the multivariate model. After univariate Cox regression analysis, all significant variables (*P* < 0.10) were entered into a stepwise forward multivariate Cox regression model. Two different models were evaluated using ECV as either a continuous or a categorical variable (ECV ≥ 33%).

A prognostic score was established in patients followed up for at least 6 months. The accuracy of ECV, risk score or LGE to predict composite outcome were evaluated by area under the receiver operator curve (ROC) curves. This score was validated in 53 consecutive HFpEF patients recruited between October 2016 and July 2017.

## Results

### Baseline characteristics

Between December 2014 and October 2016, a total of 118 consecutive HFpEF patients (78 ± 8 years, 63% women) and 26 age- and sex-matched controls (76 ± 5 years, 62% women) were included in the study. The demographic, clinical, and laboratory characteristics of HFpEF patients and controls are summarized in Table 1. We observed a high prevalence of established cardiovascular risk factors in our HFpEF population; including arterial hypertension (93%), diabetes (39%), hypercholesterolemia (67%), and higher body mass index (BMI). HFpEF patients had worse renal function and lower hemoglobin and hematocrit levels than healthy controls, and 62% of patients had a history of atrial fibrillation.

**Table 1:** Baseline characteristics of HFpEF patients and age- and sex-matched controls

	HFpEF (*n* = 118)	Healthy Controls (*n* = 26)	*P*
Age (years)	78 ± 8	76 ± 5	0.28
Body mass index (kg/m^2^)	29 ± 7	25 ± 4	0.011
Female (n, %)	74 (63)	16 (62)	0.91
Heart rate (bpm)	73 ± 14	67 ± 9	0.040
Systolic blood pressure (mmHg)	136 ± 21	144 ± 22	0.069
Diastolic blood pressure (mmHg)	75 ± 13	82 ± 12	0.014
NYHA functional class III-IV (n, %)	53 (45)	0 (0)	< 0.001
Medical history			
Atrial fibrillation (n, %)	73 (62)	0 (0)	< 0.001
Ischemic cardiomyopathy (n, %)	39 (33)	0 (0)	< 0.001
Previous valvular surgery (n, %)	12 (10)	0 (0)	0.12
Previous heart failure episode	84 (71)	0 (0)	< 0.001
Cardiovascular risk factors			
Smoking (n, %)	47 (40)	6 (23)	0.10
Hypertension (n, %)	109 (93)	16 (62)	< 0.001
Diabetes (n, %)	46 (39)	1 (4)	< 0.001
Family history of cardiovascular disease (n, %)	24 (21)	3 (12)	0.40
Hypercholesterolemia (n, %)	78 (67)	23 (88)	0.027
Medication			
Diuretics other than MRA (n, %)	94 (80)	2 (8)	< 0.001
MRA (n, %)	23 (19)	0 (0)	0.01
Beta-blockers (n, %)	76 (64)	3 (12)	< 0.001
ACE-I or ARB (n, %)	76 (64)	9 (35)	0.005
Statins (n, %)	54 (46)	5 (19)	0.01
Laboratory characteristics			
eGFR (ml/min/1.73 m^2^)	59 ± 23	70 ± 18	0.018
Hemoglobin (g/dl)	11.8 ± 1.9	14.0 ± 1.3	< 0.001
NT-proBNP (pg/ml)	1747 [374; 34,306] £	111 [29; 393] £	0.001

£ Median [min; max]

*ACE-I* angiotensin converting enzyme inhibitor, *ARB* angiotensin receptor blocker, *eGFR* estimated glomerular filtration rate; MRA:

Table 2 summarizes echocardiographic and CMR measurements. As expected, compared to age- and sex-matched healthy controls, HFpEF patients had higher E/e’ ratio, higher indexed LA and RA volumes, higher RV/RA gradient, and worse RV function as evaluated by TAPSE and FAC. Prevalence of pulmonary hypertension (RV–RA gradient > 35 mmHg) in our population was 39% (*n* = 46). By CMR, HFpEF patients had a slightly lower LVEF and higher indexed LV mass than age- and sex-matched healthy controls. Twenty-six HFpEF patients had LGE (8 = focal spots, 18 = ischemic pattern). When present, the average percentage of LGE was 5.5 ± 2.9%.

**Table 2:** Echocardiographic and CMR parameters of HFpEF patients and age- and sex-matched controls

	HFpEF (n = 118)	Healthy Controls (n = 26)	*P*
Echocardiography			
LV ejection fraction (%)	64 ± 7	64 ± 5	0.93
Transmitral peak E velocity max (m/s)	91 ± 29	55 ± 9	< 0.001
Transmitral E deceleration time (ms)	160 ± 62	197 ± 35	0.004
E/e’ septal ratio	18.1 ± 7.3	9.4 ± 1.7	< 0.001
RV/RA gradient (mmHg)	32 ± 11	19 ± 5	< 0.001
RA volume index (ml/m^2^)	35 ± 20	18 ± 5	< 0.001
RV fractional area change (%)	41 ± 9	47 ± 8	0.008
RV FAC ≤ 35% (n, %)	32 (27)	0 (0)	< 0.001
TAPSE (mm)	19 ± 5	24 ± 4	< 0.001
TAPSE≤16 mm (n, %)	47 (40)	1 (4)	< 0.001
Cardiac MR			
LVM index (g/m^2^)	68 ± 15	58 ± 12	0.003
LV EDV index (ml/m^2^)	73 ± 18	63 ± 11	0.006
LVEF (%)	63 ± 8	67 ± 5	0.024
RV EDV index (ml/m^2^)	82 ± 28	67 ± 11	0.005
RVEF (%)	57 ± 9	60 ± 6	0.052
RVEF≤45% (n, %)	16 (14)	2 (8)	0.41
LV mass/volume ratio	0.96 ± 0.20	0.94 ± 0.19	0.71
LA volume index (ml)	66 ± 29	32 ± 10	< 0.001
Myocardium native T1 time (ms)	1109 ± 82	1144 ± 47	0.038
Myocardium post contrast T1 time (ms)	353 ± 56	381 ± 64	0,028
ECV (%)	32.9 ± 4.8	28.2 ± 2.4	< 0.001
Lambda coefficient	0.52 ± 0.08	0.49 ± 0.05	0.051
ECV ≥ 33% (n, %)	49 (42)	0 (0)	< 0.001
Late gadolinium enhancement (n, %)	26 (22)	0 (0)	0.022

Values are mean ± SD. *LA* left atrium, *LV* left ventricle, *RV* right ventricle, *RA* right atrium, *BMI* body mass index

*TAPSE* tricuspid annular plane systolic excursion, *LVM* left ventricular mass, *EF* ejection fraction, *EDV* end-diastolic volume, *ECV* extracellular volume

**Fig. 1:**
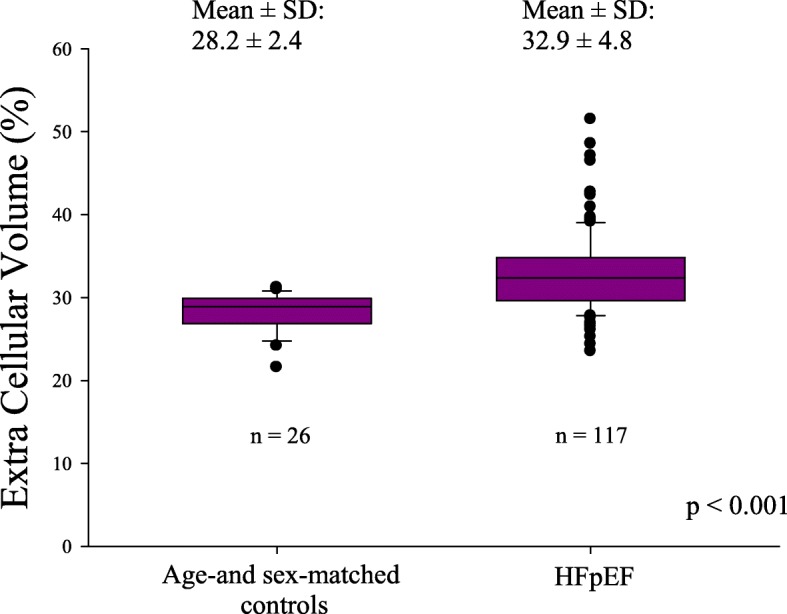
Extracellular volume fraction (ECV) values between age- and sex-matched healthy controls and heart failure with preserved ejection fraction (HFpEF) patients

ECV was significantly higher in HFpEF patients than in the healthy control group (Fig. 1). Forty-nine (42%) HFpEF patients had significant diffuse fibrosis based on the ECV cutoff (defined as ≥33%). Sixteen (14%) HFpEF patients had impaired RV systolic function defined as RVEF≤45% by CMR. HFpEF patients with RV dysfunction had higher ECV (36 ± 6% vs. 32 ± 4%, *P* < 0.001), higher indexed LA volume (81 ± 26 ml/m^2^ vs. 64 ± 28 ml/m^2^, *P* = 0.03), lower LVEF (59 ± 7% vs. 64 ± 8%, *P* = 0.010), lower TAPSE (14 ± 5 mm vs. 19 ± 5 mm, *P* < 0.001), lower FAC (32 ± 6% vs. 43 ± 8%, *P* < 0.001), and higher septal E/e’ ratio (24 ± 9 vs. 17 ± 7, *P* = 0.001).

Diabetic HFpEF patients were younger (75 ± 9 vs. 80 ± 7 years, *P* = 0.002), had a higher body mass index (BMI) (31.2 ± 6.9 vs. 27.3 ± 6.1, *P* = 0.002), a lower atrial fibrillation history rate (48% vs. 71%, *P* = 0.012), and more known ischemic cardiomyopathy (43% vs. 26%, *P* = 0.055) than non-diabetic HFpEF patients. Echocardiographic and CMR parameters were quite similar except for a trend to higher indexed LV mass in diabetics (71.0 ± 12.6 vs. 65.6 ± 16.1 g/m^2^, *P* = 0.056). Diabetic HFpEF patients presented more frequently ECV ≥33% than non-diabetic patients (52% vs. 35%, *P* = 0.061).

### Predictors of high ECV

Table 3 compares HFpEF patients with high and low ECV values. ECV and the lambda coefficient were different between the two groups, demonstrating that the difference in ECV was not solely the result of a significant difference in hematocrit level..

**Table 3:** Baseline characteristics of HFpEF patients with ECV < or ≥ 33%

	HFpEF with ECV < 33% (*n* = 68)	HFpEF with ECV ≥ 33% (*n* = 49)	*P*
Baseline characteristics			
Age (years)	78 ± 8	79 ± 9	0.49
Body mass index (kg/m^2^)	30 ± 7	28 ± 7	0.082
Female (n, %)	41 (60)	33 (67)	0.44
Systolic blood pressure (mmHg)	137 ± 21	135 ± 21	0.54
Diastolic blood pressure (mmHg)	76 ± 12	74 ± 14	0.45
NYHA functional class III-IV (n, %)	28 (41)	24 (49)	0.57
Laboratory characteristics			
eGFR (ml/min/1.73 m^2^)	58 ± 20	60 ± 28	0.56
Hemoglobin (g/dl)	12.1 ± 1.8	11.3 ± 2.0	0.020
NT-proBNP (pg/ml)	1584[432; 34,306] £	1889 [374; 27,736] £	0.40
Medical history			
Atrial fibrillation (n, %)	42 (62)	30 (61)	0.90
Ischemic cardiomyopathy (n, %)	23 (34)	16 (33)	0.94
Previous valvular surgery (n, %)	8 (12)	4 (8)	0.76
Cardiovascular risk factors			
Smoking (n, %)	29 (43)	18 (37)	0.52
Hypertension (n, %)	64 (94)	44 (92)	0.37
Diabetes (n, %)	22 (33)	24 (49)	0.069
Family history of cardiovascular disease (n, %)	14 (21)	10 (20)	0.98
Hypercholesterolemia (n, %)	47 (70)	30 (61)	0.29
Echocardiography			
Left ventricular ejection fraction (%)	64 ± 7	63 ± 8	0.66
Transmitral peak E velocity max (m/s)	86 ± 29	98 ± 26	0.025
Transmitral E deceleration time (ms)	155 ± 62	167 ± 63	0.35
E/e’ septal ratio	16 ± 6	20 ± 8	0.004
RV/RA gradient (mmHg)	31 ± 11	34 ± 11	0.13
RA volume index (ml/m^2^)	34 ± 19	36 ± 22	0.75
RV fractional area change (%)	42 ± 8	42 ± 11	0.84
TAPSE (mm)	19 ± 5	18 ± 5	0.11
Cardiac MR			
LVM index (g/m^2^)	67 ± 14	66 ± 16	0.81
LV EDV index (ml/m^2^)	71 ± 16	72 ± 20	0.58
LVEF (%)	63 ± 8	63 ± 8	0.997
RV EDV index (ml/m^2^)	77 ± 22	86 ± 32	0.097
RVEF (%)	56 ± 7	56 ± 10	0.98
RVEF≤45% (n, %)	6 (9)	10 (20)	0.073
LV mass/volume ratio	0.97 ± 0.21	0.94 ± 0.19	0.43
LA volume index (ml/m^2^)	65 ± 29	68 ± 28	0.68
Myocardium native T1 time (ms)	1109 ± 83	1107 ± 82	0.88
Myocardium post contrast T1 time (ms)	362 ± 58	340 ± 52	0.036
ECV (%)	30.0 ± 2.2	37.0 ± 4.3	< 0.001
Lambda coefficient	0.48 ± 0.05	0.57 ± 0.08	< 0.001
Late gadolinium enhancement	13 (19)	13 (28)	0.29

Values are mean ± SD. £: median (min, max); *LA* left atrium, *LV* left ventricle, *RV* right ventricle, *RA* right atrium, *BMI* body mass index, *TAPSE* tricuspid annular plane systolic excursion, *LVM* left ventricular mass, *EF* ejection fraction, *EDV* end-diastolic volume, *ECV* extracellular volume

HFpEF patients with ECV ≥ 33% had lower BMI, lower hematocrit, higher prevalence of diabetes, higher transmitral peak E wave velocity and higher E/e’ ratio. A higher proportion of HFpEF patients with ECV ≥ 33% had an impaired RV systolic function (CMR RVEF ≤45% (20% vs. 9%, *P* = 0.073). There were no differences in NT-proBNP and LGE in HFpEF patients between the two groups.

In multivariate logistic regression, BMI (OR = 0.92 [0.86–0.98], *P* = 0.011), presence of diabetes (OR = 2.62 [1.11–6.18], *P* = 0.028), and higher transmitral peak E wave velocity (OR = 1.02 [1.00–1.03], *P* = 0.022) were significantly associated with high ECV value.

### Outcomes

**Fig. 2:**
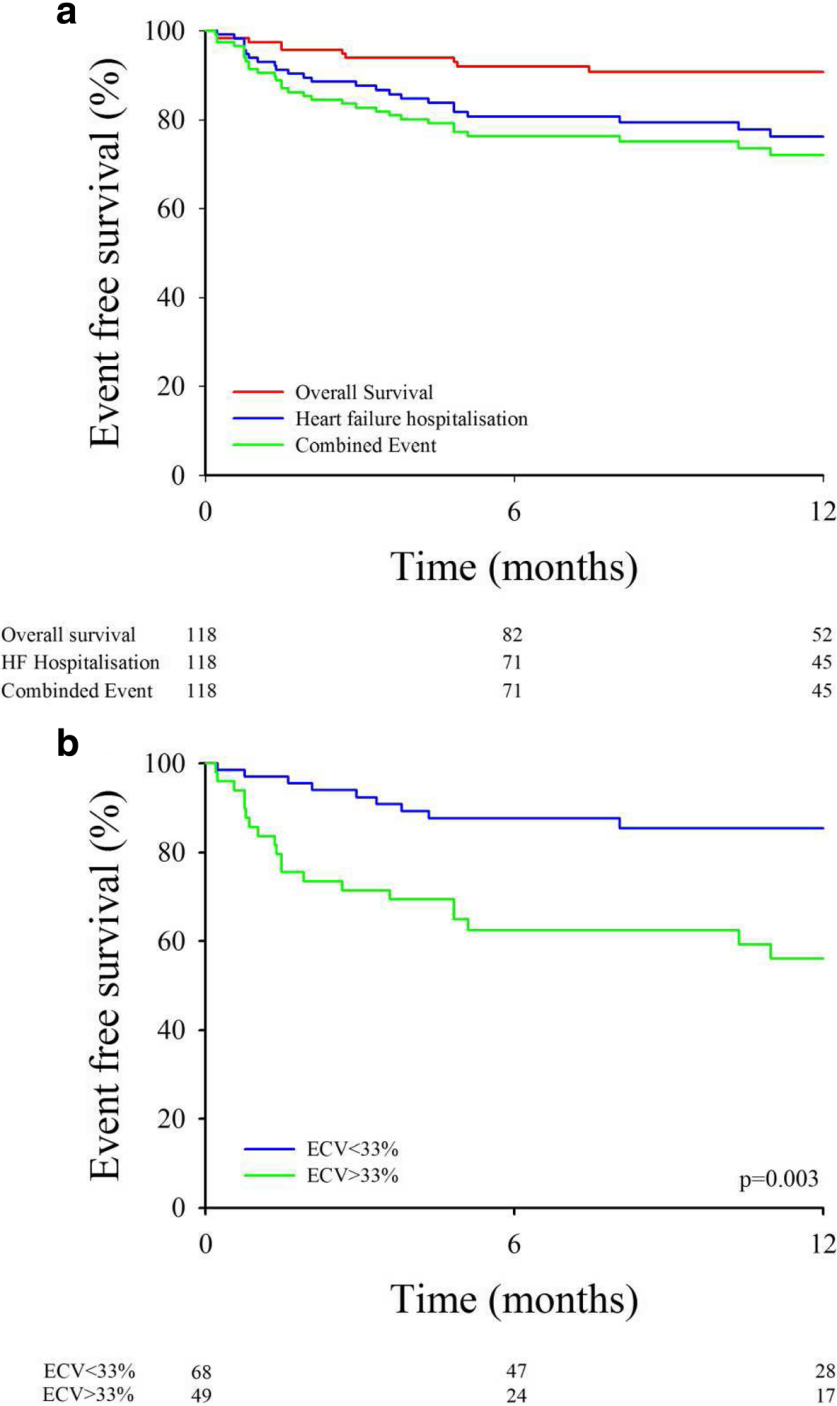
(**a**) Mortality and HF hospitalization Kaplan Meier Curves (**b**) Event-free survival in HFpEF patients according to ECV cutoff

During a mean follow-up of 11 ± 6 months, we observed 43 events (11 deaths and 32 hospitalizations for HF) (Fig. 2a). The primary outcome (all-cause mortality or first hospitalization for HF) occurred in 38 patients (32%). Only one patient was lost to follow up. The percentage of combined event at 18 months in our population was 50%.

In univariate Cox regression analysis, lower eGFR, lower hemoglobin, presence of diabetes, higher transmitral E wave velocity, and ECV ≥ 33% were significant predictors of the composite outcome (Table 4). In multivariate Cox regression analysis, only the presence of diabetes (HR = 1.98 [1.04; 3.76], *P* = 0.038) and hemoglobin level (HR = 0.81 [0.67; 0.98], *P* = 0.028) were significantly associated with the composite outcome (Table 5). The ability of either ECV as a continuous variable or ECV as a categorical variable (ECV > 33%) to improve this model was then tested and added significant prognostic information (χ^2^ 4.33, *P* = 0.037 and χ^2^ 4.46, *P* = 0.035, respectively), as opposed to transmitral peak E wave velocity and eGFR (χ^2^ 3.00, *p* = 0.083 and χ^2^ 2.86, *p* = 0.091 respectively). The ability of the model to discriminate outcome was good with an area under the curve (AUC) of 0.76. Kaplan–Meier event-free survival curves showed that HFpEF patients with ECV ≥ 33% had poorer one-year prognosis than those with ECV < 33% (56 ± 8% vs 82 ± 5%, *P* = 0.001, Fig. 2b).

**Table 4:** Univariate Cox analysis for composite event (death and first HF)

	Composite outcome (death or first HF)	
	HR [95%IC]	*P*
Age (years)	1.00 [0.96; 1.04]	0.96
Body surface area (m^2^)	0.29 [0.08; 1.08]	0.065
Female (n, %)	1.63 [0.79; 3.36]	0.19
Heart rate (bpm)	1.02 [1.00; 1.04]	0.086
Systolic blood pressure (mmHg)	0.99 [0.97; 1.01]	0.18
Diastolic blood pressure (mmHg)	0.97 [0.95; 1.00]	0.064
NYHA functional class III-IV	1.18 [0.62; 2.26]	0.62
Laboratory characteristics		
eGFR (ml/min/1.73 m^2^)	0.98 [0.96; 0.99]	0.040
Hemoglobin (g/dl)	0.80 [0.66; 0.96]	0.020
NT-proBNP (pg/ml)	1.12 [0.82; 1.55]	0.48
Medical history		
Atrial fibrillation (n, %)	1.15 [0.58; 2.25]	0.69
Ischemic cardiomyopathy (n, %)	1.03 [0.52; 2.04]	0.94
Previous valvular surgery (n, %)	1.16 [0.35; 3.83]	0.81
Previous heart failure episode	1.17 [0.50; 2.73]	0.72
Cardiovascular risk factors		
Smoking (n, %)	0.99 [0.51; 1.92]	0.98
Hypertension (n, %)	0.81 [0.25; 2.68]	0.73
Diabetes (n, %)	2.15 [1.13; 4.08]	0.020
Family history of cardiovascular disease (n, %)	0.97 [0.45; 2.12]	0.94
Hypercholesterolemia (n, %)	1.04 [0.51; 2.11]	0.91
Echocardiography		
LV ejection fraction (%)	1.03 [0.98; 1.08]	0.20
Transmitral peak E velocity max (m/s)	1.01 [1.00; 1.02]	0.025
E/e’ septal ratio	1.02 [0.98; 1.06]	0.42
RV/RA gradient (mmHg)	1.01 [0.98; 1.04]	0.60
RA volume index (ml/m^2^)	1.00 [0.99; 1.02]	0.53
RV fractional area change (%)	2.01 [0.06; 73.25]	0.70
TAPSE (mm)	0.94 [0.89; 1.00]	0.064
Cardiac MR		
LVM index (g/m^2^)	0.99 [0.98; 1.01]	0.62
LV EDV index (ml/m^2^)	0.99 [0.98; 1.01]	0.58
LVEF (%)	1.03 [0.99; 1.07]	0.19
RV EDV index (ml/m^2^)	1.00 [0.99; 1.01]	0.59
RVEF (%)	0.99 [0.96; 1.04]	0.81
LV mass/volume ratio	1.24 [0.06; 25.3]	0.89
LA volume index (ml/m^2^)	1.00 [0.98; 1.02]	0.87
Myocardium native T1 (ms)	1.01 [0.99; 1.01]	0.23
ECV (%)	1.07 [1.01; 1.12]	0.015
ECV ≥ 33%	2.62 [1.35; 5.09]	0.005
LGE (%)	1.08 [0.96; 1.21]	0.22

**Table 5:** Multivariate Cox analysis for event-free survival in HFpEF

	HR ([95% CI]	*P*	Χ^2^ to remove	Χ^2^ to enter	*P*
Diabetes mellitus	1.98 [1.04; 3.76]	0.038	4.327		
Hemoglobin	0.81 [0.67; 0.98]	0.028	4.961		
Mean E wave				3.00	0.083
eGFR				2.86	0.091
Model 1: ECV (continuous variable)	1.07 [1.00; 1.13]			4.33	0.037
Model 2: ECV 33%	2.00 [1.00; 4.03]			4.46	0.035

*CI* confidence interval

A prognostic score was established based on the significant predictors of composite outcome in patients with at least 6 months of follow up (*n* = 97). Diabetes, hemoglobin, and ECV > 33% were thus used to build the risk score. ROC curves showed a better discrimination with the prognostic score and ECV (c-statistic of 0,76 and 0,67 respectively) compared to LGE (c statistic 0,51) (Additional file 1). In the validation cohort of 53 patients, 2 deaths and 9 HF hospitalizations were observed during a mean follow up of 11 ± 5 months.

Our risk score based on the initial cohort and applied in the validation cohort had an AUC of 0.71 to predict primary outcome.

## Discussion

We sought to evaluate the presence, associations, and prognostic significance of quantification of ECV using 3 T CMR in a prospective and well-characterized HFpEF cohort. The salient findings of our study are as follows. Mean ECV by T1-mapping was significantly higher in HFpEF patients than in age-matched healthy controls; ECV in HFpEF patients was related to the presence of anemia and diabetes and associated with altered diastolic function by echocardiography and lower CMR RV systolic function; and finally, increased ECV and the presence of anemia and diabetes were independent risk markers of short-term poor prognosis with increased rehospitalization or all-cause mortality in HFpEF.

### HFpEF

HFpEF is a heterogeneous disease observed mainly in the aging population. We and others demonstrated that aging is associated with structural changes in the heart, such as alterations of diastolic function, increased stiffening, LV and atrial remodeling characterized by decreased LV and RV volumes and mass, increased atrial volumes, and an increase in ECV [21]. It is crucial to separate age-related changes from disease-related processes associated with HFpEF. Therefore, we compared our population to carefully age- and sex-matched healthy subjects, allowing for a better understanding of these two processes. As compared to previous work [22, 23], our HFpEF population was older and had more comorbidities, probably reflecting a less selected population but a more advanced stage because patients were highly symptomatic with high NT-proBNP levels (45% NYHA III/IV). Overall, and in accordance with prior reports, we observed relatively poor outcomes with a high rehospitalization rate and high mortality despite optimal medical treatment.

### Role of extracellular matrix abnormalities in HFpEF

Extracellular matrix abnormalities causing LV stiffening and secondary LV diastolic dysfunction are probably among the main potential pathophysiologic mechanisms involved in HFpEF. Previous studies using endomyocardial biopsies or autopsies have already shown that the extent of fibrosis is higher in HFpEF patients than in controls [7].Studies have demonstrated that ECV closely correlates with histologically determined diffuse interstitial fibrosis, providing a non-invasive estimation for its quantification; however, only a few studies have evaluated ECV in HFpEF patients [15, 22, 23]. Our findings confirm that high ECV likely reflecting diffuse extracellular matrix abnormalities, a potential surrogate for myocardial fibrosis, may play a key role in the pathophysiology of HFpEF. Indeed, ECV was significantly increased relative to the age-matched population, yet only 42% of our HFpEF patients had high ECV, suggesting that other mechanisms are involved in HFpEF pathophysiology. Assessment of ECV allows for a direct evaluation of the extracellular space, reflecting interstitial disease. However, it lacks the ability to provide information about the relative contribution of edema, fibrosis, inflammation, or deposition of other extracellular proteins such as amyloid [13] and therefore may not allow for a full understanding of the pathophysiological mechanisms underlying HFpEF. In our study, low BMI, diabetes, and high transmitral peak E wave velocity were significant determinants of high ECV. High transmitral peak E wave velocity is a good surrogate for high filling pressures, and it could be easily anticipated as an important determinant of fibrosis.

Surprisingly, we observed that a higher BMI was associated with lower ECV values, suggesting that diffuse fibrosis is not the sole mechanism involved in HFpEF pathogenesis in the obese population. Indeed, adiposity-induced inflammation has considerable adverse effects, including endothelial dysfunction, capillary rarefaction, and mitochondrial dysfunction in both the cardiac and systemic beds [24].

Diabetic cardiomyopathy could be considered as a form of HFpEF, explaining the increased incidence of HF in the diabetic population [25]. This cardiomyopathy is characterized by insulin resistance and a loss of metabolic flexibility, and subsequently by cardiomyocyte hypertrophy and increased fibrosis. Increased reactive oxygen species production is one of the major pathophysiological mechanisms triggered by hyperglycemia and high free fatty acid level.

Finally, native T1 time was surprisingly significantly lower in HFpEF patients than in healthy controls. We can only speculate on the explanation. Possible explanation could be that HFpEF patients have slightly higher intramyocadial iron concentration or more intramyocardial fat, particularly related to presence of diabetes.

### Prognosis in HFpEF

As demonstrated in our study, the prognosis of HFpEF patients is still quite poor. Many studies have compared HFpEF and HFrEF prognosis and demonstrated the same or even a worse mortality and morbidity rate [26, 27] for HFpEF than for HFrEF. Our study identified high ECV, hemoglobin and diabetes as independent prognostic predictors for the short-term composite outcome. This result is in accordance with observations by Redfield et al. [28], who showed that profibrotic pathways may contribute to adverse outcomes in diabetic HFpEF patients. However, in our work, presence of LGE was not a significant predictor of the composite outcome (HR = 1.08 [0.96; 1.21], *p* = 0.22) in univariate analysis, in contrast to another recent study [16]. A potential explanation was that patients in this pre cited study had significantly higher percentage of LGE quantification (13 ± 8%), as opposed to our population where only 26 patients had small amounts of LGE (5.5 ± 2.9%).

The prognostic role of anemia has already been demonstrated in large HFpEF studies (SENIOR [29], MAGGIC [30], ARIC [26], respectively).

### Clinical implications

Because HFpEF syndrome is a heterogeneous disease, better characterization of HFpEF phenotypes based on clinical presentation and biological and/or imaging data is crucial for better designing therapies [31]. In particular, the identification of anemia is relevant because its association with composite outcome could suggest a potential beneficial effect of iron-replacement therapy in HFpEF patients, and this hypothesis could be evaluated in larger randomized trials. Our study contributes to a better understanding of the heterogeneous and complicated nature of HFpEF [32]. Larger studies are needed to confirm our findings and prospectively validate our risk markers in other populations.

### Study limitations

Our study is a single-center study of relatively small size, and its power is limited by a modest number of events, which bears a risk of overfitting in multivariable models. Yet our study was prospective, and the echocardiographic and CMR imaging, as well as biomarker sampling, were standardized. Because we excluded patients with contraindications to CMR, in particular renal failure, our conclusions cannot be generalized to all patients with HFpEF. Although we validated ECV assessment by T1 mapping in other populations against histopathology [15] with reasonably good correlation, in this fragile elderly population, we did not sample cardiac biopsies and thus could not ascertain the pathophysiological correlates of increased ECV in our patients. Another limitation is the lack of others SAX slices for the MOLLI acquisition to have a better idea of ECV value in the rest of the myocardium.

Focal fibrosis is probably also very important in HFpEF patients but only 26 patients had LGE.

Moreover, we interpreted CMR data from patients in atrial fibrillation but the impact of atrial fibrillation on MOLLI sequences has not been fully studied yet.

In addition, our study was performed mainly in a white population, and findings might differ in other groups, particularly in African American or other populations. Finally, the presence of different comorbidities is an important confounding factor in HFpEF. Thus, findings might be affected by selection criteria and presence of comorbidities in such HFpEF populations.

## Conclusions

Among HFpEF patients, high ECV likely reflecting increased myocardial fibrosis was associated with BMI, diabetes, and transmitral peak E wave velocity. Among HFpEF patients, abnormal diffuse myocardial fibrosis estimated by ECV was associated with a higher rate of all-cause death and first HF hospitalization in the short term follow up.

## Additional file

Additional file 1: Risk score. (DOCX 62 kb)

## Abbreviations

2D: Two dimensional
AUC: Area under the curve
BMI: Body mass index
bSSFP: Balanced steady state free precession
CMR: Cardiovascular magnetic resonance
ECG: Electrocardiogram
ECV: Extracellular volume fraction
EDV: End-diastolic volume
EF: Ejection fraction
eGFR: Estimated glomerular filtration rate
ESV: End-systolic volume
FAC: Fractional area change
HF: Heart failure
HFpEF: Heart failure with preserved ejection fraction
HFrEF: Heart failure with reduced ejection fraction
LA: Left atrium/left atrial
LV: Left ventricle/left ventricular
LVM: Left ventricular mass
MOLLI: Modified Look-Locker inversion recovery
NYHA: New York Heart Association
RA: Right atrium/right atrial
ROC: Receiver operator curve
ROI: Region-of-interest
RV: Right ventricle/right ventricular
SAX: Short axis
TAPSE: Tricuspid annular plane systolic excursion
