# Supplementary material for: Associations and prognostic significance of diffuse myocardial fibrosis by cardiovascular magnetic resonance in heart failure with preserved ejection fraction
Source: J Cardiovasc Magn Reson. 2018 Aug 8;20:55. doi: 10.1186/s12968-018-0477-4 (PMC6081897; doi:10.1186/s12968-018-0477-4)

**Additional file 1**

**Risk score**

We tried to phenotype our HFpEF patients thanks to a risk score built on the 3 parameters associated with combined outcome. Diabetes, hemoglobin, and ECV33% were then used to develop a risk score.

**Statistics analysis**

This score was established in patients followed up for at least 6 months. (n=96). Therefore, a logistic regression model was built using predictive variables from multivariate Cox regression. From this score, we derived the corresponding probability of risk to present the event based on the inverse of the logistic function (exp(risk score)/(1+exp(risk score)).

We determine a hemoglobin cut off value with a ROC curve (Hemoglobin 11g/dl). The Area under the curve was 0.64 with a sensitivity of 0.55% and specificity of 0.75%.

To validate this score, we first did an internal validation thanks to the one-leave estimation method. Then, we did an extern validation of our risk score in another validation cohort of 53 HFpEF patients. With a Chi square test, we compared event rate in the tertiles of the two cohorts.

**Results**

Diabetes, hemoglobin, and ECV33% were used to build the risk score. The following equation was obtained: -1.7+ 1.2*Diabetes+ 0.6*ECV33 + 1.3*Hb11g. From this score, we derived a corresponding probability of risk of having an event. Kaplan–Meier analysis revealed a significant difference among the three different groups separated according to the tertile of probability score (p<0,001, Fig. 1).

The accuracy of ECV, risk score or LGE to predict composite outcome were evaluated by area under the ROC curves.ROC curves showed a better discrimination with the prognostic score and ECV (c-statistic of 0,76 and 0,67 respectively) compared to LGE (c statistic 0,51). (Fig 2)

Finally, we validated our score. First, we made an intern validation on the 96 initial patients, by the one leave estimation method, where 86% of patients were well classified. Then, we used the validation cohort of 53 HFpEF patients (age 77±8years, 47% women). 2 deaths and 9 HF hospitalizations were observed during a mean follow up of 11±5 months. Our risk score had AUC of 0.71 in this cohort. We observed the same proportion of events in the different tertiles, in the initial and in the validation cohort. With a Chi square test, we compared the tertiles of the two cohorts, and there was no statistical difference (tertile 1: p=0.054; tertile 2: p=0.084; tertile 3: p=0.38).

However larger studies are needed to confirm our findings and prospectively validate our risk markers in other populations.

Figure S1. Combined outcome according to a prognostic score. (a) Kaplan–Meier curve. (b) Examples.

|  | **Patient 1** | **Patient 2** | **Patient 3** |
| --- | --- | --- | --- |
| ECV (%) | 33.6 | 30.4 | 27.8 |
| Diabetes | 1 | 0 | 0 |
| Hemoglobin | 10.8 | 10.7 | 12.5 |
| Risk score (based on the formula) | 1.34 | -0.44 | -1.70 |
| Probability of having an event (%) | 79 | 39 | 15 |

Figure S2. ROC curves comparing the prognostic value of LGE, ECV and the prognostic score.


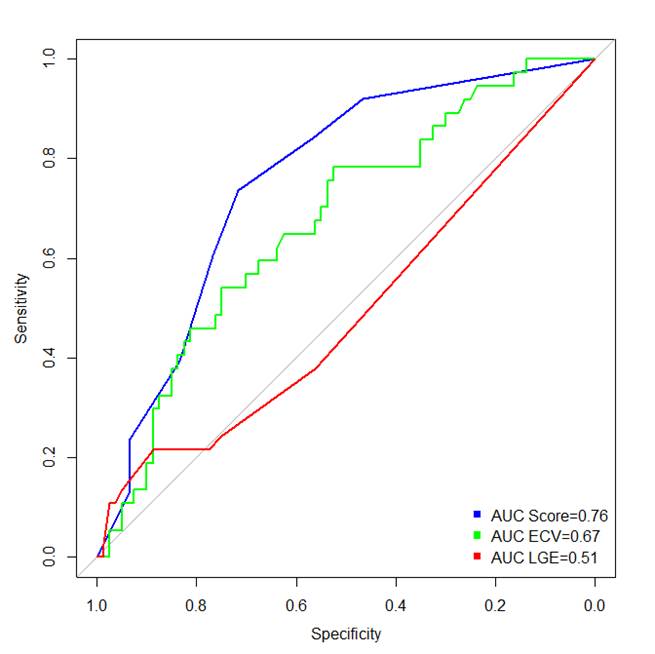

Supplement: Supplementary file 1 — Risk score. (DOCX 62 kb) [file 12968_2018_477_MOESM1_ESM.docx]
